# Dihydroartemisinin-Piperaquine vs. Artemether-Lumefantrine for First-Line Treatment of Uncomplicated Malaria in African Children: A Cost-Effectiveness Analysis

**DOI:** 10.1371/journal.pone.0095681

**Published:** 2014-04-18

**Authors:** Johannes Pfeil, Steffen Borrmann, Yeşim Tozan

**Affiliations:** 1 Centre for Childhood and Adolescent Medicine and Department of Infectious Diseases, University Hospital, Heidelberg, Germany; 2 German Centre for Infection Research (DZIF), Heidelberg, Germany; 3 Kenyan Medical Research Institute (KEMRI), Kilifi, Kenya; 4 Institute of Tropical Medicine, University of Tübingen, Tübingen, Germany; 5 Steinhardt School of Culture, Education and Human Development, New York University, New York, New York, United States of America; 6 Institute of Public Health, Ruprecht-Karls-University, Heidelberg, Germany; Tulane University School of Public Health and Tropical Medicine, United States of America

## Abstract

**Background:**

Recent multi-centre trials showed that dihydroartemisinin-piperaquine (DP) was as efficacious and safe as artemether-lumefantrine (AL) for treatment of young children with uncomplicated *P. falciparum* malaria across diverse transmission settings in Africa. Longitudinal follow-up of patients in these trials supported previous findings that DP had a longer post-treatment prophylactic effect than AL, reducing the risk of reinfection and conferring additional health benefits to patients, particularly in areas with moderate to high malaria transmission.

**Methods:**

We developed a Markov model to assess the cost-effectiveness of DP versus AL for first-line treatment of uncomplicated malaria in young children from the provider perspective, taking into consideration the post-treatment prophylactic effects of the drugs as reported by a recent multi-centre trial in Africa and using the maximum manufacturer drug prices for artemisinin-based combination therapies set by the Global Fund in 2013. We estimated the price per course of treatment threshold above which DP would cease to be a cost-saving alternative to AL as a first-line antimalarial drug.

**Results:**

First-line treatment with DP compared to AL averted 0.03 DALYs (95% CI: 0.006–0.07) and 0.001 deaths (95% CI: 0.00–0.002) and saved $0.96 (95% CI: 0.33–2.46) per child over one year. The results of the threshold analysis showed that DP remained cost-saving over AL for any DP cost below $1.23 per course of treatment.

**Conclusions:**

DP is superior to AL from both the clinical and economic perspectives for treatment of uncomplicated *P. falciparum* malaria in young children. A paediatric dispersible formulation of DP is under development and should facilitate a targeted deployment of this antimalarial drug. The use of DP as first-line antimalarial drug in paediatric malaria patients in moderate to high transmission areas of Africa merits serious consideration by health policymakers.

## Introduction

Despite a rapid scaling up of malaria control efforts and recent reports of decreasing transmission intensities in African countries, malaria remains an important cause of morbidity and mortality, particularly in young children [1]. The choice of first-line antimalarial drug for treatment of uncomplicated *Plasmodium falciparum* malaria is critical in preventing the progression of acute infections to severe disease and reducing the risk of further morbidity, disability and premature mortality from the disease [2]. Artemisinin-based combination therapies (ACTs) are recommended by the World Health Organization (WHO) for first-line treatment of uncomplicated *P. falciparum* malaria worldwide [3]. Artemether-lumefantrine (AL, Coartem®, Novartis Pharma AG), a fixed-dose co-formulated ACT, is the most widely deployed antimalarial drug in African countries today [4].

Recent multi-centre trials showed that dihydroartemisinin-piperaquine (DP), a newer fixed-dose co-formulated ACT, was as efficacious and safe as AL for treatment of young children with uncomplicated malaria across diverse transmission settings in Africa [5], [6]. Longitudinal follow-up of patients in these trials supported previous findings that DP had a longer post-treatment prophylactic effect than AL, reducing the risk of reinfection and conferring additional health benefits to patients, particularly in areas with moderate to high malaria transmission where reinfections are common [7]–[10]. DP has recently been added to WHO's list of recommended ACTs and is considered a promising candidate for first-line treatment of uncomplicated malaria [3].

Significant financial resources have been put into scaling up the access to ACTs through global subsidies on manufacturing prices of drugs [11]. The maximum manufacturer drug prices for ACTs have recently been set by the Global Fund; the price per course of treatment in children ranges between $0.43–$1.22 for AL and $0.66–$0.93 for DP [12]. We used these newly negotiated drug prices and assessed the cost-effectiveness of DP versus AL for first-line treatment of uncomplicated malaria in young children, taking into consideration the post-treatment prophylactic effects of these two leading ACTs over a follow-up period of 63 days reported by a randomized, non-inferiority clinical trial of four different ACTs in Africa [6]. The trial was conducted at 12 sites across seven countries between 2007 and 2009 where local transmission rates ranged from meso-endemic seasonal to high perennial with entomological inoculation rates (EIR) of 60 to over 563 infective bites per person per year. One site was characterized as an area with low perennial transmission with an EIR of 6 infective bites per person per year. The analysis adopted the provider perspective in order to inform decision-making on antimalarial treatment policies at the health systems level.

## Methods

**Figure 1:**
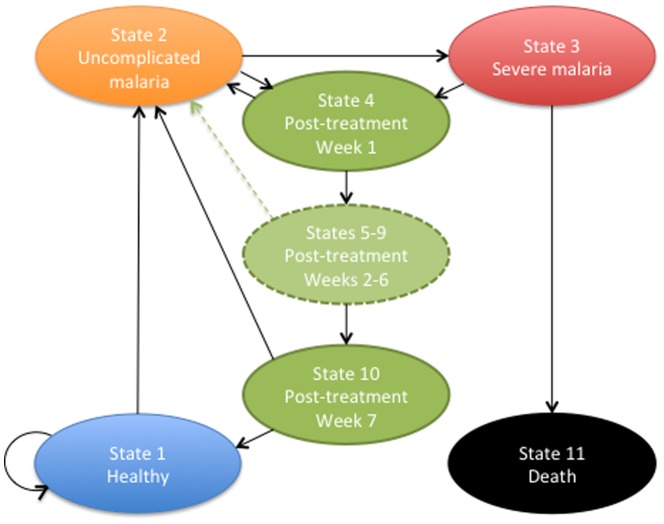
Illustration of the Markov model. Children enter the Markov model in the “healthy” state. Healthy children can either remain healthy, or acquire uncomplicated malaria according to the weekly hazard rate of uncomplicated malarial disease. Children in the “uncomplicated malaria” state receive first-line treatment with either DP or AL, and they either recover fully (recovers to post- treatment) or progress to severe malaria (acquires severe malaria). Children in the “severe malaria” state either recover fully or with permanent neurologic sequelae and enter the “post-treatment” state for a period of seven weeks, or die (dead). Children in the “post-treatment state” can either remain free of recurrent malaria (continues post-treatment), or get re-infected (acquires uncomplicated malaria) according to the weekly hazard rates for recurrent malaria depending on the post-treatment prophylactic effects of DP and AL. Children who remain free of recurrent malaria seven weeks after treatment enter the “healthy” state.

Cost-effectiveness analysis was performed using a Markov model (TreeAge Pro 2014, TreeAge Software Inc., MA, USA) that defined a set of mutually exclusive health states, simulating the progression of malarial disease and the risk of recurrent malaria in children younger than five years of age, receiving DP or AL for first-line treatment of uncomplicated malaria (figure 1). The Markov model was run in weekly cycles over a period of one year. We estimated the mean incremental costs and health outcomes of the two treatment strategies over this period, and all results are presented per child. Incremental cost-effectiveness ratios (ICERs) were reported when a treatment strategy was not ruled out of the decision analysis by simple dominance [13]. Incremental costs and ICERs were calculated in United States dollars ($) for the year 2013. All input parameters, their distributions, and data sources are listed in Table 1.

**Table 1:** Markov model input variables (all costs are in US dollars for the year 2013).

Input variable	Distribution	Distribution parameters
Transition probabilities		
Hazard rate for uncomplicated malarial disease in susceptible children^a^	Beta	0.0467 (SD 0.0036)
Weekly hazard rate for recurrent malaria following first-line treatment with DP^a^		
Week 1	Beta	0.0305 (SD 0.0045)
Week 2	Beta	0.0203 (SD 0.0038)
Week 3	Beta	0.0150 (SD 0.0033)
Week 4	Beta	0.0246 (SD 0.0042)
Week 5	Beta	0.0892 (SD 0.0081)
Week 6	Beta	0.0644 (SD 0.0072)
Week 7	Beta	0.0663 (SD 0.0076)
Weekly hazard rate for recurrent malaria following first-line treatment with AL^a^		
Week 1	Beta	0.0220 (SD 0.0042)
Week 2	Beta	0.0117 (SD 0.031)
Week 3	Beta	0.0228 (SD 0.044)
Week 4	Beta	0.1347 (SD 0.0108)
Week 5	Beta	0.1707 (SD 0.0131)
Week 6	Beta	0.0542 (SD 0.0081)
Week 7	Beta	0.0585 (SD 0.0086)
Proportion of treated uncomplicated cases progressing to severe malaria [22]	Beta	0.03 (SD 0.0170)
Proportion of severe malaria survivors having persisting NS^b^ [15], [16]	Beta	0.00995 (α = 27; β = 2,686)
Case fatality rate for severe malaria after inpatient care^b^ [15], [16]	Beta	0.109 (α = 297; β = 2,416)
Uncomplicated malaria treatment		
DP cost per course of treatment [12]	Uniform	0.66–0.93
AL cost per course of treatment [12]	Uniform	0.43–1.22
Average duration of illness (days) (assumed)	Point estimate	2
Severe malaria treatment (inpatient care)		
Cost of drugs per child [20]	Uniform	Min-max 3.65–4.90
Cost of diagnostic investigations per child [20]	Uniform	Min-max 6.98–31.31
Cost of hospital bed-day [20]	Point estimate	11.52
Average length of hospital stay (days) when patient recovers fully [23]	Triangle	Mode 4.5 (min-max 3–7)
Average length of hospital stay (days) when patient recovers with neurological sequelae [22]	Point estimate	10
Average length of hospital stay (days) when patient dies (assumed)	Point estimate	2

DP = Dihydroartemisinin Piperaquine; AL = Artemether-Lumefantrine; SD = Standard Deviation; Max = Maximum; Min = Minimum.

a Using the data reported by a multi-centre trial of ACTs on the number of patients whose treatment was failure free (N) over a follow-up period of 63 days [6], the weekly hazard rates for recurrent malaria following treatment with DHPQ and AL were estimated using Kaplan-Meier estimator as h_t_ = 1−(N_t_/N_t-1_), where t = 1, 2,…9 weeks. The hazard rate for uncomplicated malarial disease in healthy children was estimated by taking the average of the hazard rates for recurrent malaria at weeks 8 and 9 following treatment with DP and AL.

b For these clinical outcomes, β distributions were calculated based on the incidence of mortality and neurological sequelae in malaria patients as reported in a randomized trail that compared parental treatment with either artesunate or quinine in African children with severe malaria [15], [16].

### Markov model and transition probabilities

The Markov model had 11 health states: State 1: Healthy; State 2: Uncomplicated malaria; State 3: Severe malaria; States 4–10: Post-treatment from week one to week seven; and State 11: Dead (figure 1). The post-treatment states were characterized as tunnel states (a set of temporary states that must be visited only in a fixed sequence) [13] in the Markov model and represented the first seven weeks following oral antimalarial treatment during which a patient was at risk of recurrent malaria, caused by re-infection. The use of tunnel states allowed the model to incorporate the time-dependent nature of the post-treatment prophylactic effect of DP and AL and define the experience of patients following treatment. The transition probabilities in the post-treatment states were estimated as weekly hazard rates for recurrent malaria, using the Kaplan-Meier estimator and the proportion of patients whose treatment was failure free over a period of 63 days reported by the randomized, non-inferiority clinical trial of four different ACTs (table 1) [6]. We assumed that any difference in the weekly hazard rates for recurrent malaria between the two treatment groups would vanish seven weeks after treatment, and all treated children would become susceptible to disease based on the level of malaria endemicity. We estimated the hazard rate for uncomplicated malarial disease in susceptible children by taking the average of the estimated weekly hazard rates for recurrent malaria at weeks 8 and 9 (table 1).

Published estimates were used for the probability of developing severe malaria following oral antimalarial treatment [14], the case fatality rate of severe malaria following inpatient care, and the proportion of severe malaria survivors developing persisting neurological sequelae [15], [16] (table 1). These figures correspond to areas with moderate to high malaria transmission.

### Estimating health outcomes

Children entered the Markov model in the “healthy” state. They were subjected to a hazard rate for unscomplicated malarial disease estimated from the trial data and flowed through the subsequent health states in weekly cycles. We assumed that all children with uncomplicated malaria would be treated promptly with either DP or AL, and all children with severe malaria would receive inpatient care. Children, who were treated orally with antimalarial drugs, would either recover and enter the “post-treatment” states, or develop severe malaria. Depending on the antimalarial treatment choice, children in the post-treatment states were subjected to a weekly hazard rate for recurrent malaria during the first seven weeks following treatment (table 1). Children who remain free of recurrent malaria seven weeks after treatment enter the “healthy” state. Children in the “severe malaria” state either recover fully or with permanent neurologic sequelae and enter the “post-treatment” state for a period of seven weeks, or die (dead).

Health outcomes were measured in terms of disability adjusted life years (DALYs) averted. DALYs combine years of life lost because of premature mortality with years of life lived with disability. We used an average life expectancy of 57.25 years for children aged 1–4 years on the basis of the life tables for African men and women for the WHO sub-region with high child and high adult mortality [17]. The disability weights for treated uncomplicated cases and treated neurological sequelae were set at 0.211 and 0.436, respectively [18]. DALYs were discounted at 3%, as recommended by the World Bank [19]. Age weighting was not applied; a year of healthy life was valued equally at all ages. We also estimated the number of uncomplicated and severe malaria cases and the number of deaths per child over one year for both treatment strategies.

### Estimating costs

We considered the costs of oral antimalarial drugs for treating uncomplicated cases and the costs of inpatient care for severe cases over one year (table 1), but excluded the costs of illness accruing to patients because of the assumed provider perspective. The price per course of treatment was $0.66 and $0.93 for children receiving 3 times 20/160 mg and 3 times 40/320 mg DP tablets, respectively, and $0.43, $0.83 and $1.22 for children receiving 6 times 20/120 mg, 6 times 40/240 and 6 times 60/360 mg non-dispersible AL, respectively.

We used published estimates of inpatient care costs at primary level hospitals from a recent Kenyan costing study [20] and adjusted the reported costs in 2005 for the year 2013, using the consumer price index [21]. Drug and diagnostic investigation costs per child ranged between $3.65–$4.90 and $6.98–$31.31, respectively. Costs per hospital stay per patient were calculated per day per hospital bed at a rate of $11.52 [20]. The average length of hospital stay varies according to the health outcome of the patient. The average length of hospital stay is 4.5 days if the patient had a full recovery and is 10 days if the patient recovered with neurologic sequelae [22], [23]. The majority of deaths from malaria occur within 24–48 hours of hospital admission. Hence we assumed an average hospital stay of 2 days if the patient died.

### Sensitivity analysis

**Figure 2:**
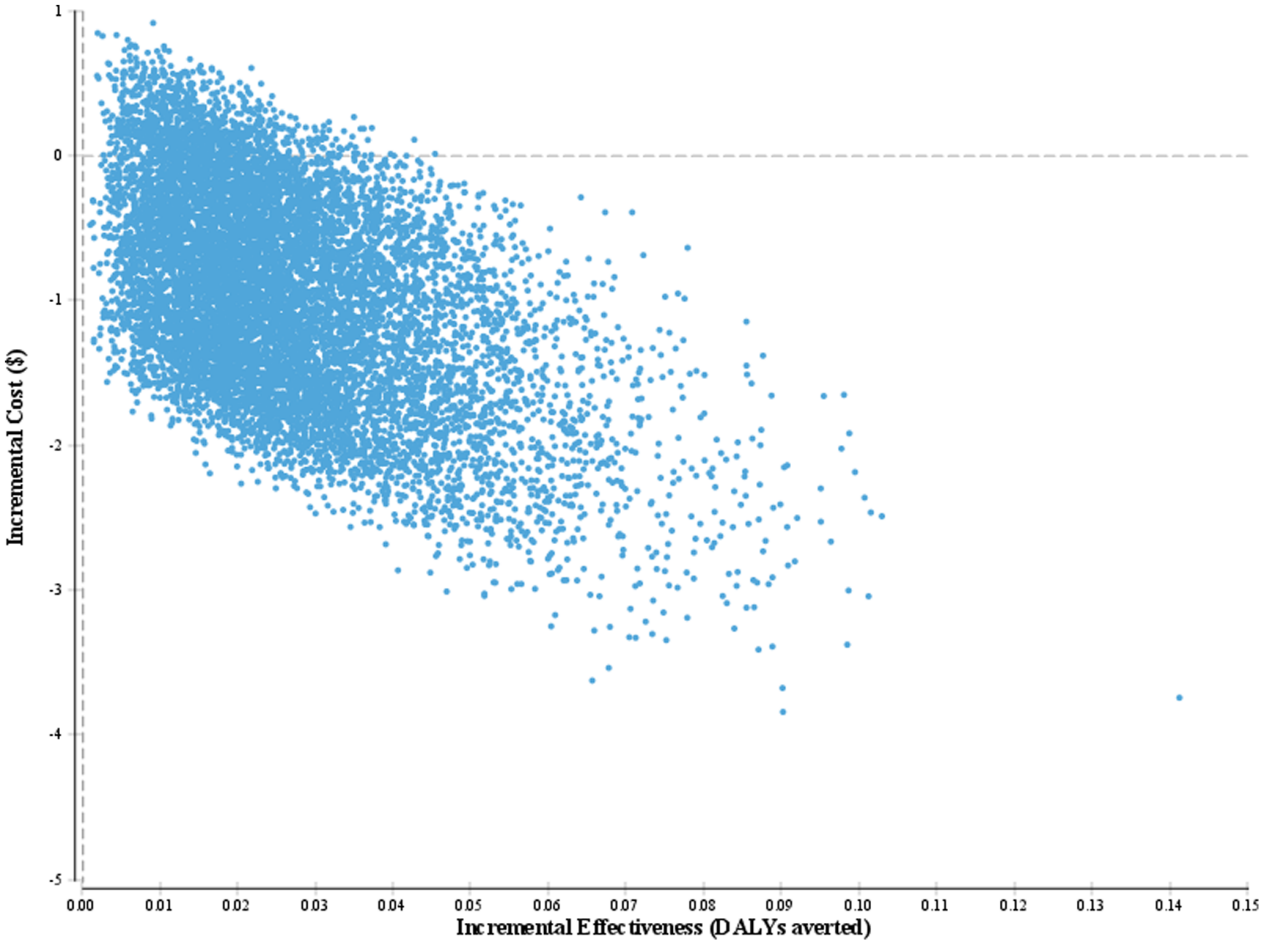
Scatterplot of incremental costs and effectiveness for DP vs. AL for first-line treatment of uncomplicated *P. falciparum* malaria in young children based on probabilistic sensitivity analysis (10,000 iterations) (all values are calculated per child over one year). DP = Dihydroartemisinin-Piperaquine; AL = Artemether-Lumefantrine; DALY = Disability-Adjusted Life Year.

To assess the uncertainty in key model parameters and the robustness of the results to model assumptions, we performed a probabilistic sensitivity analysis using a Monte Carlo simulation technique with 10,000 iterations. For each iteration, a value for each input variable was selected randomly from its distribution given in table 1. Results of the probabilistic sensitivity analysis were presented as a scatter plot of incremental costs and health outcomes (figure 2). We also undertook a threshold analysis to determine the cost per course of treatment above which DP would cease to be a cost-saving alternative to AL for first-line treatment of uncomplicated malaria. The cost of DP per course of treatment was varied between $0.5 and $3.0, while holding all other input variables at their mean values.

## Results

Table 2 shows the mean costs and effectiveness of first-line treatment with DP and AL as well as the mean differences in costs and effectiveness with 95% confidence intervals (CI); all values were calculated per child over one year. The cumulative mean number of uncomplicated malaria cases was 2.25 (95% CI: 2.00–2.50) and 2.55 (95% CI: 2.27–2.83) per child when treated with DP and AL, respectively. The cumulative mean number of severe malaria cases was 0.07 (95% CI: 0.01–0.16) and 0.08 (95% CI: 0.02–0.18) per child when treated with DP and AL, respectively. The cumulative mean number of deaths was estimated to decline from 0.008 (95% CI: 0.001–0.021) to 0.007 (95% CI: 0.001–0.019) per child when AL is substituted with DP as first-line antimalarial drug.

**Table 2:** Results of cost-effectiveness analysis: all values are calculated per child over one year (all costs are in US dollars for the year 2013).

	First-line treatment with DP Mean (95% CI)	First-line treatment with AL Mean (95% CI)	Incremental outcomes: DP vs. AL Mean (95% CI)
Cases of uncomplicated malaria	2.25 (2.00–2.50)	2.55 (2.27–2.83)	0.30 (0.20–0.40)
Cases of severe malaria	0.07 (0.01–0.16)	0.08 (0.02–0.18)	0.01 (0.002–0.02)
Deaths	0.007 (0.002–0.017)	0.008 (0.002–0.019)	0.001 (0.00–0.002)
DALYs	0.21 (0.05–0.50)	0.24 (0.05–0.56)	0.03 (0.006–0.07)
Costs	6.88 (2.77–14.52)	7.85 (3.00–16.43)	−0.96 (−2.46–0.33)

CI = Confidence Interval; DALY = Disability-Adjusted Life Year.

First-line treatment with DP was the economically dominant treatment strategy over AL with a 90% probability (figure 2), resulting in a mean improvement of 0.03 DALYs (95% CI: 0.006–0.07) and 0.001 deaths (95% CI: 0.00–0.002) averted per child and a mean cost saving of $0.96 (95% CI: 0.33–2.46) per child over one year (table 2).

## Discussion

This is the first economic analysis of first-line treatment options for uncomplicated *P. falciparum* malaria that considered the post-treatment prophylactic effects of antimalarial drugs – an important secondary benefit of antimalarial treatment, particularly in areas with moderate to high malaria transmission in Africa. DP was both clinically superior and less costly compared to AL for first-line treatment of uncomplicated malaria in young children. It proved to be the economically dominant treatment strategy in such transmission settings. The threshold analysis showed that first-line treatment with DP would remain cost saving over AL for any DP cost below $1.23 per course of treatment.

Our analysis is based on the primary data from a cohort of children who participated in a multi-centre trial on the efficacy of ACTs conducted in areas with moderate to high malaria transmission in Africa [6]. The Markov model estimated an annual incidence rate of 2.25 (95% CI: 2.00–2.50) and 2.55 (95% CI: 2.27–2.83) cases of uncomplicated *P. falciparum* malaria per child when treated with DP and AL, respectively. A recent study predicted an annual incidence rate of 1.7 (95% CI: 1.4–3.9) cases of uncomplicated malaria per child for this age group living in such transmission settings in Africa [1], while another longitudinal study in a high transmission area reported 4.82 and 4.61 treatments per person-year in Ugandan infants treated with DP or AL, respectively [24]. Our results are consistent with the published estimates, and the model provides a realistic estimate of malaria morbidity in this age group. From a clinical and economic perspective, the benefits of post-treatment prophylaxis are expected to become more significant with increasing transmission intensity and, conversely, less significant with decreasing transmission intensity.

We assumed full access to treatment with AL and DP and same level of patient compliance to both therapies to evaluate the costs and benefits conferred by the post-treatment prophylactic effects of the drugs. While both drugs are administered over three days, DP has a simple, once daily dosing regimen whereas AL should be given twice daily and ideally with a fatty meal. Therefore, DP has the potential to improve patient compliance and treatment effectiveness and hence overall management of pediatric patients. However, such an advantage of treatment with DP could not be evaluated in a clinical trial with an observed drug intake [6] and was not factored into our analysis, potentially underestimating the effectiveness of first-line treatment with DP in usual care settings. On the other hand, the trial reported that in children treated with AL gametocyte prevalence during follow-up and gametocyte carriage time were significantly lower than in children treated DP. However, it is unclear how this would affect the intensity of malaria transmission in a locality over time.

A limitation of our analysis is the lack of data on the long-term post-treatment prophylactic effect of DP compared to AL. Our model incorporated data from a drug efficacy trial with limited (63 days) longitudinal follow-up of patients [6]. We assumed that the prophylactic effect of both DP and AL would vanish seven weeks after treatment, setting a lower bound for our results by underestimating the effectiveness of first-line treatment with DP compared to AL. This assumption is supported by the long terminal half-life of piperaquine (2–3 weeks) and the reported re-infection rates by the multi-centre trials in Africa [5], [6]. However, it might be the case that slowly eliminated antimalarial drugs may suppress reinfection to a time point beyond the relatively short follow-up periods of trials. By assuming equal hazard rates for uncomplicated malarial disease in both treatment groups from seven weeks onwards, we might have overestimated the prophylactic capacity of treatment with DP.

Maximum manufacturer prices are negotiated by the Global Fund for several ACTs, including AL and DP. However, these prices do not necessarily reflect the first-line buyer prices; co-payment amounts go up to 98% of manufacturer sales prices for some African countries [12]. We did not use these heavily subsidized drug prices in this analysis because co-payment agreements are in effect for only six African countries, and co-payment amounts vary widely across these countries. Furthermore, co-payment percentages apply similarly to both AL and DP in any given country.

It should be mentioned that a change in national malaria treatment policies may require substantial resources [25]. A Tanzanian study reported that the cost of changing the first-line antimalarial treatment from chloroquine to sulfadoxine–pyrimethamine was $0.02 per person [25]. The cost savings from substituting AL with DP as first-line antimalarial drug have the potential to offset some of these costs. Nevertheless, incremental costs and incremental health outcomes reported in this analysis represent a range of best estimates, and the study findings apply to a target population of young children living in moderate to high transmission settings. Semi-immunity, which is normally found in older children and adults, infections with non-falciparum species, or lower *P. falciparum* endemicity will affect the efficacy and the post-treatment prophylactic effect of these drugs. Decision makers should contextualise costs and assess patient compliance, pattern of clinical malaria, and other key parameters in their own settings to arrive at more locally representative results.

There is limited evidence on the cost-effectiveness of ACTs for treatment of uncomplicated malaria in children. A recent study from Papua New Guinea compared the cost-effectiveness of three different ACTs against the conventional treatment with chloroquine plus SP for treatment of *P. falciparum* (and *P.vivax*) malaria [26]. The main outcome of the study was the cost per treatment success, which was defined as “adequate parasitological and clinical response over a 42 day follow-up period.” Polymerase chain reaction (PCR) correction was applied to distinguish recrudescent parasites from new infections. The authors concluded that AL was the most effective treatment regimen, followed by DP, and both ACTs were highly cost-effective for treatment of *P. falciparum* malaria in children compared to the conventional treatment. From a patient's perspective, the differentiation between recrudescence and re-infection is insignificant. PCR-correction discounts the post-treatment prophylactic effect of a drug by excluding new infections that occur during the follow-up period. Large-scale clinical trials in African settings have consistently shown that DP was more efficacious than AL using PCR-uncorrected cure rates [5], [6]. By assessing the impact of post-treatment prophylaxis on costs and health outcomes, our study adds a new perspective on the optimal drug choice for first-line treatment of uncomplicated *P. falciparum* malaria in children in areas with moderate to high transmission.

A main concern with DP and other long-acting antimalarial drugs is that residual drug levels play a critical role in the emergence and spread of drug resistance [27]. While piperaquine has been shown to linger at ineffective concentrations for months in very young children and may potentially promote the spread of piperaquine-tolerant parasites [28], this has not been confirmed in pharmacodynamic studies. It has been suggested that the benefit of a short-acting drug to cure an initial infection might be outweighed by its inability to reduce the risk of re-infection in moderate to high transmission settings [9]. Therefore, this should not be a deterrent for large-scale deployment of DP, but rather stresses the urgent need for effective surveillance systems that would allow for early detection of resistance to antimalarial drugs, which also shortens the period of post-treatment prophylaxis [29].

There is an urgent need to improve the management of malaria in African children. Decision makers who aim to provide optimal treatment strategies to the populations at risk need to consider various aspects of antimalarial drugs – including safety, tolerability, dosing schedule, level of drug resistance, post-treatment prophylactic effect, and cost – and malaria endemicity. Our study demonstrates the superiority of DP over AL from the clinical and economic perspectives for treatment of uncomplicated *P. falciparum* malaria in young children. A paediatric dispersible formulation of DP suitable for use by children between the ages of six months and five years is under development [30] and can facilitate targeted administration of the drug to this age group. The use of DP as first-line antimalarial drug for paediatric malaria patients in moderate to high transmission areas of Africa merits serious consideration by health policy makers.
